# A conjugated polymer antimicrobial agent triggers metabolic cascade-mediated killing of carbapenem-resistant *Klebsiella pneumoniae*

**DOI:** 10.3389/fcimb.2026.1796479

**Published:** 2026-04-16

**Authors:** Shuyun Wang, Song Wang, Lin Chen, Binbin Li, Chuangxin Zhang, Donghong Yin, Jinju Duan

**Affiliations:** 1 Department of Pharmacy, Second Hospital of Shanxi Medical University, Taiyuan, Shanxi, China; 2 School of Pharmacy, Shanxi Medical University, Taiyuan, Shanxi, China; 3 School of Chemistry and Chemical Engineering, Shanxi University, Taiyuan, Shanxi, China

**Keywords:** activity-tunable, compensatory, conjugated, CRKP, metabolic hijacking, polymer, self-destruction, spatiotemporal control

## Abstract

**Introduction:**

Carbapenem-resistant *Klebsiella pneumoniae* (CRKP) skin infections represent a critical therapeutic challenge due to biofilm formation and poor drug penetration. Reactive oxygen species (ROS)-mediated strategies, such as advanced photodynamic therapy, offer promising multi-target approaches against such resistant pathogens.

**Methods:**

We developed a light-tunable conjugated polymer, P3, designed to penetrate CRKP biofilms and achieve spatiotemporally controlled activation. Its efficacy was evaluated against established CRKP biofilms in both dark and visible light conditions, assessing biofilm biomass elimination and bacterial viability.

**Results:**

P3 leveraged its intrinsic optical properties and ROS production to eliminate 50% of the CRKP biofilm in darkness. Upon visible light irradiation, its efficacy was dramatically enhanced via a triggered compensatory self-destruction (CSD) mechanism. This self-amplifying reaction catastrophically disrupted bacterial membrane integrity and oxidative balance, resulting in the internal destruction of over 80% of the biofilm and bacterial death. This action combines physical matrix breakdown with ROS-mediated biomolecular damage, leading to near-complete biofilm eradication.

**Discussion:**

Our study demonstrates that P3 functions as a "metabolic time bomb," providing a targeted, antibiotic-free strategy against structured bacterial communities. These findings highlight the significant potential of this light-controlled platform for treating resistant biofilm-associated infections.

## Introduction

*Klebsiella pneumoniae* (KP) is an opportunistic Gram-negative pathogen that colonizes human mucosal surfaces and can cause severe infections (e.g., pneumonia, bacteremia) in immunocompromised hosts (Martin and Bachman, 2018; Yang et al., 2021; Liu et al., 2024). In China, KP is the third most common cause of wound infections (11.4%), as reported by CHINET-2023, trailed only by E. coli and S. aureus (CHINET, 2023). The rampant misuse of antibiotics drives the evolution of multidrug-resistant K. pneumoniae (MDR-KP), selecting for mechanisms including carbapenemase (e.g., KPC, NDM) production, efflux pump overexpression, and biofilm formation. This has culminated in the emergence of carbapenem-resistant K. pneumoniae (CRKP), a major public health threat evidenced by high resistance rates to imipenem (24.8%) and meropenem (26.0%) (The changes in resistance of Klebsiella pneumoniae to imipenem and meropenem; Fang et al., 2020; Chi et al., 2022).

While classic skin pathogens like Staphylococcus aureus remain clinically important, the therapeutic options against them are relatively broader. In stark contrast, the extreme drug resistance of CRKP has created a dire and specific therapeutic void. This challenge is particularly acute in the context of skin and soft tissue infections (SSTIs), where an already compromised skin barrier and poor drug penetration are compounded by CRKP’s formidable resistance mechanisms. These factors frequently lead to persistent, biofilm-associated infections with severely limited treatment options (Liu et al., 2014; Al-Wrafy et al., 2022; Baker et al., 2023). Consequently, CRKP-associated SSTIs are notoriously difficult to manage, as biofilm formation, a key resistance mechanism, creates structured bacterial communities within a protective extracellular polymeric substance (EPS) matrix that severely limits antimicrobial penetration and efficacy (Flemming and Wingender, 2010; Li et al., 2013; Su et al., 2022). Given that about 91.2% of KP isolates form biofilms, there is an urgent need for novel agents with potent, penetrative anti-CRKP and antibiofilm activity, acting via non-traditional mechanisms (Bado et al., 2016; Khodadadian et al., 2018).

PDT is a promising alternative against drug-resistant pathogens due to its targeted action and low resistance risk (Schastak et al., 2010; Maisch, 2015; Tuchin et al., 2022). Clinically applied in conditions like skin ulcers and periodontitis, PDT employs a photosensitizer (PS) activated by specific-wavelength light to generate cytotoxic reactive oxygen species (ROS). These ROS induce irreversible oxidative damage to essential bacterial components such as lipids, nucleic acids, and proteins, leading to cell death (Xia et al., 2023). A key advantage of PDT is its spatiotemporal control, meaning the therapeutic action (ROS generation) is confined both spatially to the illuminated area and temporally to the duration of light exposure. This enables targeted eradication of multidrug-resistant pathogens with minimal systemic toxicity (Liu et al., 2015). However, its efficacy against biofilms is limited by poor photosensitizer (PS) penetration through the dense EPS matrix. Notably, the inherent negative surface charge of both bacterial membranes and EPS can be leveraged by cationic agents. Quaternary ammonium compounds, for example, exploit this electrostatic attraction to disrupt biofilm integrity and enhance PS penetration, thereby addressing a key constraint of PDT (Wu et al., 2022; Wu et al., 2022; Wang et al., 2023).

Cationic conjugated polymers (CCPs) are advanced photosensitizers for antibacterial PDT, combining efficient, tunable ROS (e.g., singlet oxygen) generation with inherent cationic groups that promote binding to negatively charged bacterial and biofilm surfaces (Zhu et al., 2012; Nguyen et al., 2022). Feng et al. synthesized a fluorene-co-phenylene CCP, P3, whose optical and ROS-generating properties were tuned via intramolecular charge transfer by modifying its π-conjugated backbone. Introducing quaternary ammonium side chains enhanced its water solubility and bacterial interaction. When the P3 concentration was 5 μm, P3 demonstrated efficacy against ampicillin-resistant E. coli and low cytotoxicity under light, attributed to combined ROS generation and cationic action (Wang et al., 2019). As both CRKP and E. coli are Gram-negative with an LPS outer membrane, P3 may overcome carbapenemase-mediated resistance via charge-driven membrane penetration and ROS-mediated oxidation. However, CRKP’s thick capsule and robust biofilm could impede P3 delivery, potentially limiting its activity.

To address this challenge and investigate its potential to overcome the key clinical barriers of biofilm penetration and multidrug resistance, we utilized clinically isolated CRKP strains as a rigorous model. We aimed to thoroughly assess the antibacterial and antibiofilm activity of P3, its penetration capability, and the underlying mechanisms. The ultimate goal is to develop a groundbreaking, localized therapeutic strategy for difficult-to-treat CRKP infections such as chronic wounds and biofilm-contaminated medical devices.

## Results

### Antibacterial activity of P3 *in vitro*

**Figure 1:**
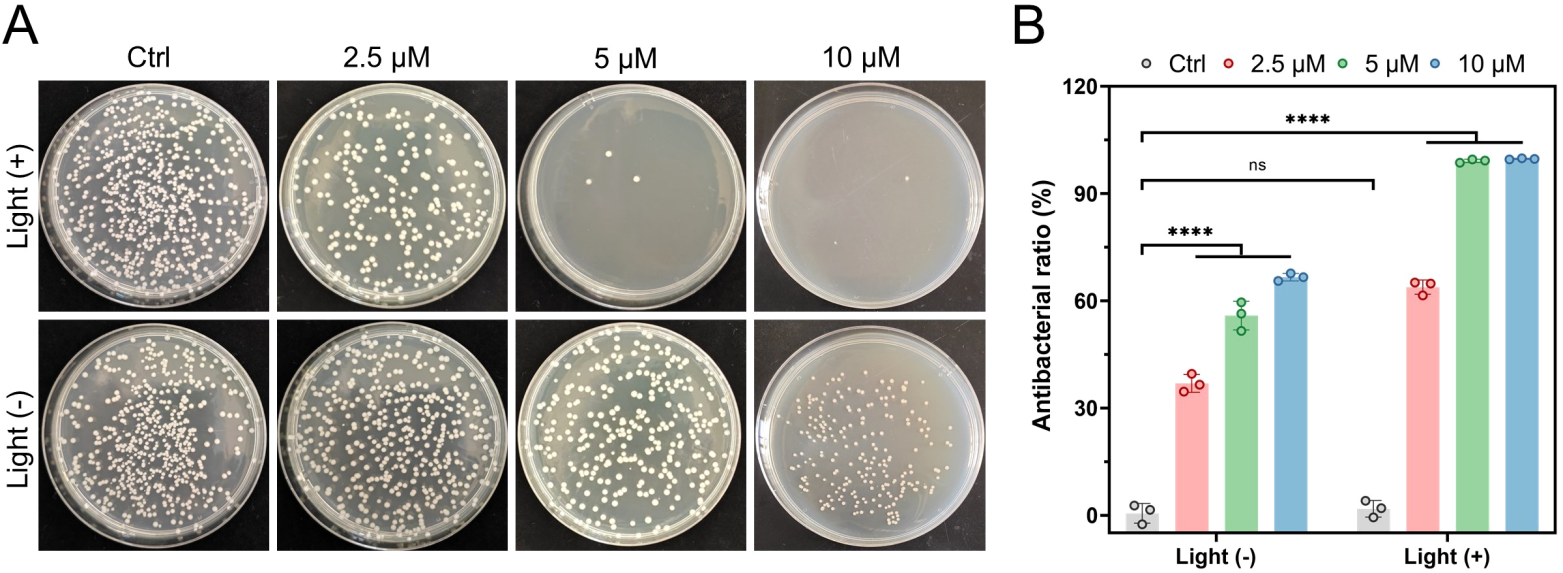
Activity of P3 Against CRKP *In Vitro*. **(A)** Colony plate photographs of P3 against CRKP. **(B)** Bactericidal rate of P3. ****P<0.0001, ns expressed P value > 0.05, with no significance.

According to the plate count method, P3 exhibited concentration-dependent antibacterial activity under dark conditions (2.5, 5, and 10 μM: 36.9%, 55.8%, and 66.6%, respectively). Following light exposure, activity markedly increased to 63.8%, 99.1%, and 99.7% at the same concentrations, corresponding to relative increases of 72.8%, 77.5%, and 49.7% (**Figure 1**). The *in vitro* results confirm that P3 possesses significant, light-enhanced, and dose-dependent antibacterial effects.

### Antibacterial mechanism of P3 *in vitro*

#### SEM and TEM analysis of bacterial morphological and intracellular changes

**Figure 2:**
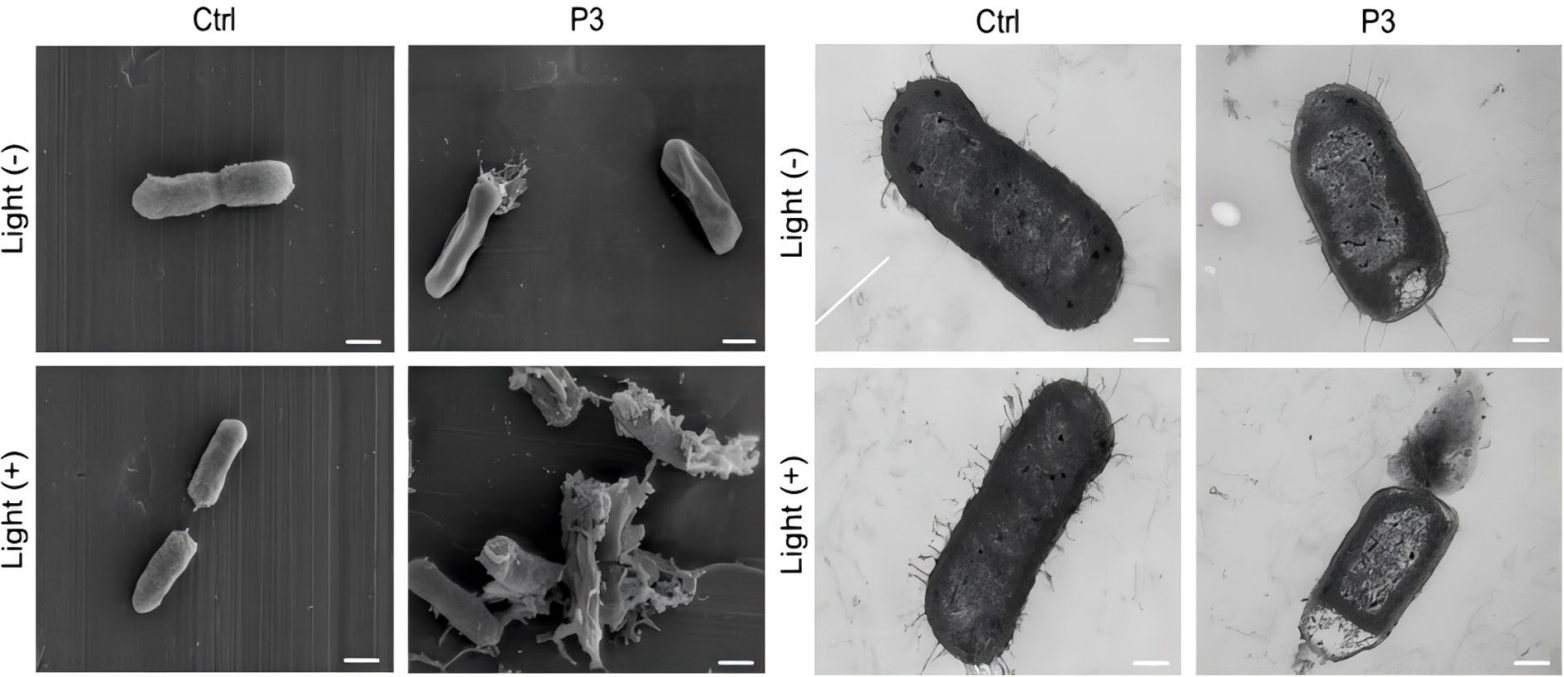
SEM and TEM images of P3 Against CRKP. Scale bar: 500 nm and 250 nm, respectively.

The control group of CRKP was treated with PBS. Under SEM, these cells exhibited clear morphology, intact surfaces, and were ready for division. TEM analysis showed that the bacteria were structurally intact, with densely packed and homogeneous cytoplasm. In contrast, SEM analysis of CRKP treated with P3 revealed mild membrane damage, and TEM also showed a significant degradation of cytoplasmic density and a reduction in the number of flagella. When CRKP was treated with P3 plus light, SEM analysis showed notable membrane damage, including depression and rupture; this resulted in the leakage of cytoplasmic contents into the surrounding environment. TEM observations during this treatment revealed flagellar detachment, extensive membrane damage, a decrease in cytoplasmic density accompanied by vacuole formation, and the extrusion of cellular materials from the cell poles (**Figure 2**).

#### Protein leakage assay and ROS detection

**Figure 3:**
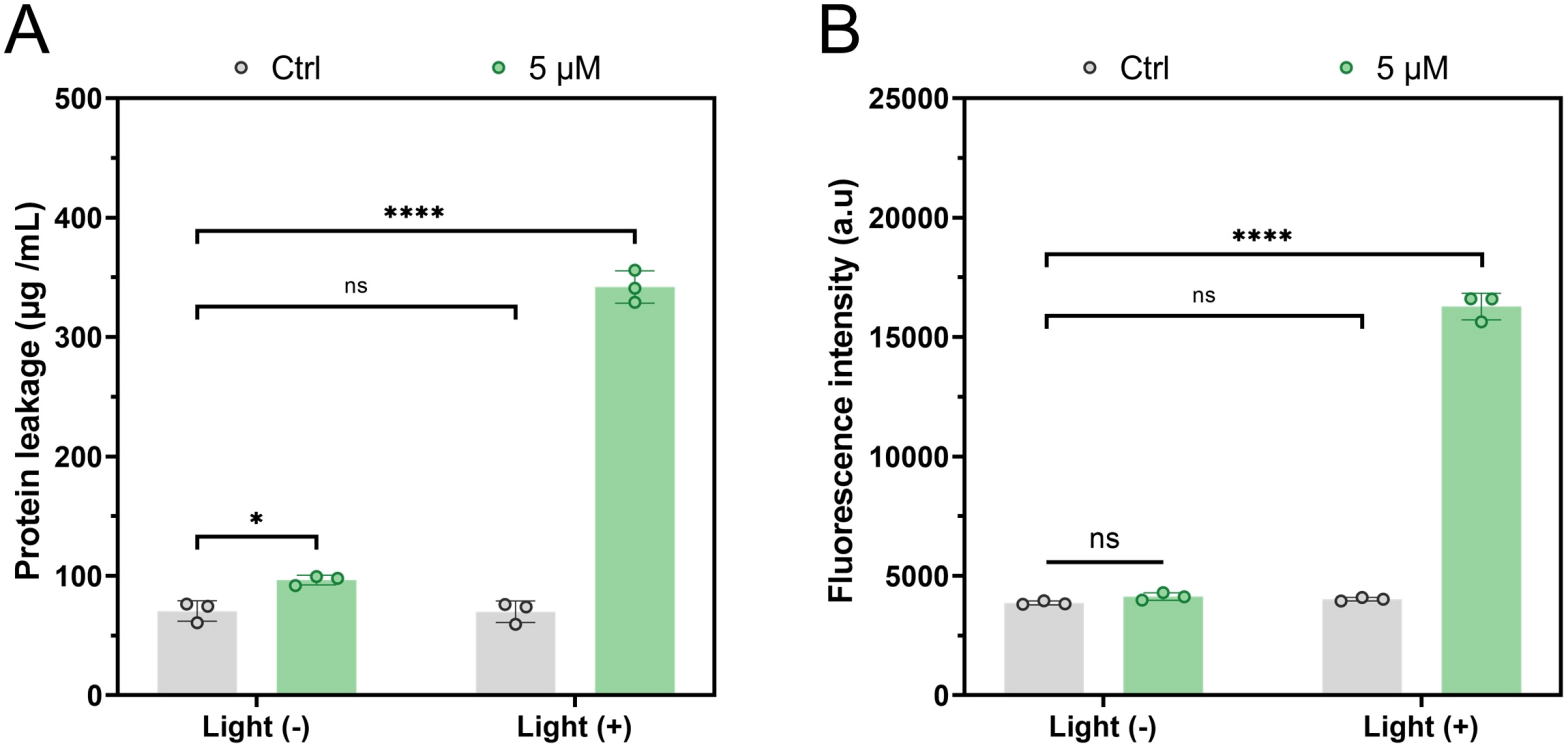
Protein Leakage **(A)** and DCF Fluorescence Intensity **(B)** across Experimental Conditions. ****P<0.0001, ns expressed P value > 0.05, with no significance.

As shown in **Figure 3A**, protein leakage assays revealed that P3-treated CRKP released significantly more protein under dark conditions (96.51 ± 4.04 μg/mL) than the control group (70.59 ± 8.56 μg/mL; P< 0.05). Moreover, in the light, P3-treated CRKP exhibited a markedly enhanced protein release (341.97 ± 13.50 μg/mL), compared to the control CRKP (69.97 ± 9.03 μg/mL; P< 0.0001). ROS generation of the two groups were quantified with the DCFH-DA assay. After alkaline hydrolysis to DCFH, the probe is oxidised to fluorescent DCF by ROS. In the dark, comparable levels of DCF fluorescence were observed in the P3-treated and control groups (3867.95 ± 80.10 a.u. vs. 4137.06 ± 157.29 a.u.), indicating baseline ROS production associated with P3. In contrast, upon light exposure, P3 treatment induced substantial ROS generation, resulting in an approximately four-fold increase in DCF fluorescence (16,279 ± 559 a.u. vs. 4,027 ± 74 a.u.; P< 0.0001; **Figure 3B**).

#### Biofilm eradication capability

**Figure 4:**
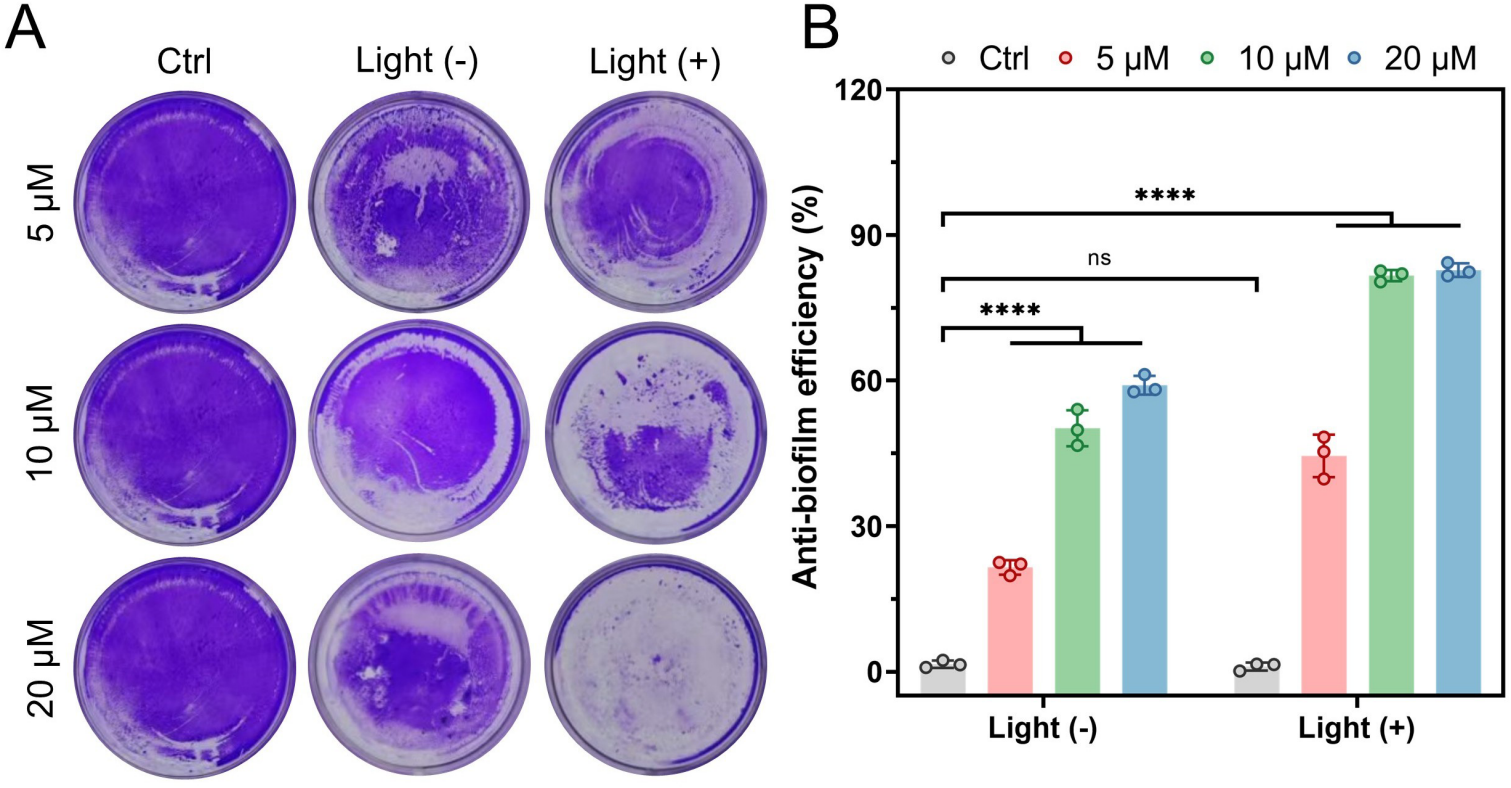
**(A)** Crystal Violet Staining of P3-Treated Biofilms. **(B)** Quantitative Analysis of P3-Treated Biofilms. ****P<0.0001, ns expressed P value > 0.05, with no significance.

The anti-biofilm efficacy of P3 was concentration and light dependent. Under dark conditions, P3 (5, 10, 20 μM) reduced biofilm biomass by 21.5%, 50.2%, and 59.0%, respectively. Light irradiation significantly enhanced eradication, achieving 44.5%, 81.7%, and 82.8% reduction, representing a 107.0%, 62.7%, and 40.3% increase over corresponding dark treatments (**Figures 4A, B**).

**Figure 5:**
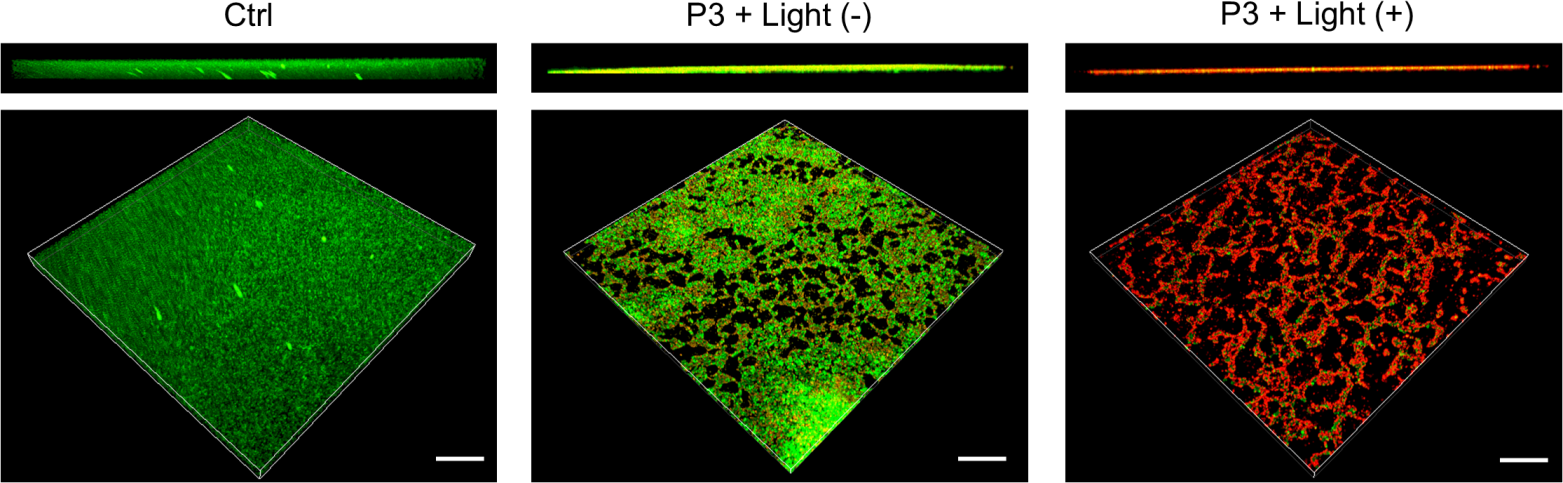
Live/dead staining of P3-treated CRKP biofilms, scale bar: 50 μm.

Calcein-AM/PI staining showed control biofilms were thick and structurally intact with dense green fluorescence, indicating high viability. P3 treatment reduced biofilm thickness and induced sporadic red fluorescence, suggesting membrane disruption and cell death (**Figure 5**).

**Figure 6:**
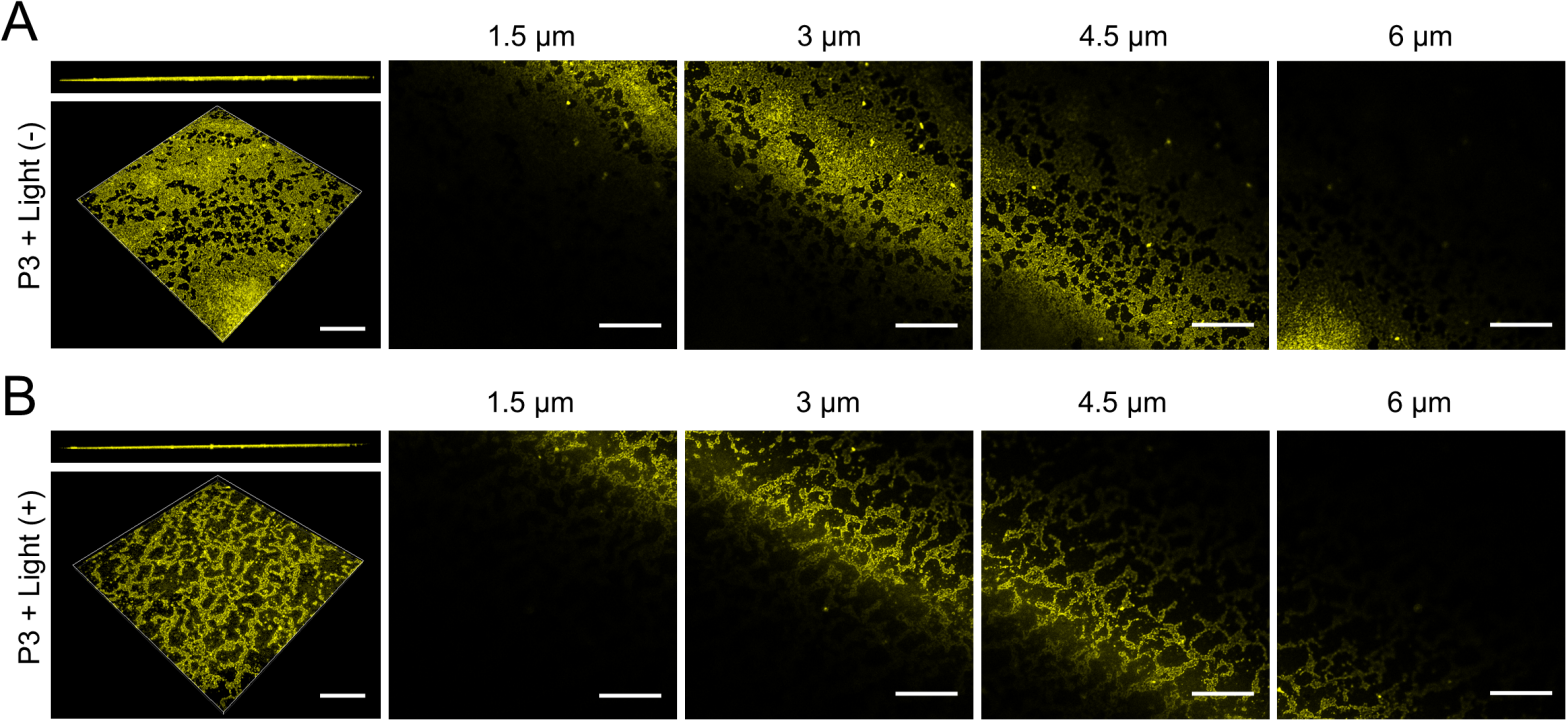
Biofilm penetration by P3. **(A)** P3 + Light(-); **(B)** P3 + Light(+). Scale bar: 50 µm.

The intrinsic fluorescence of P3 demonstrated deep penetration (80% depth) into biofilms during incubation, with uniform distribution throughout the matrix. Subsequent light activation triggered biofilm dissociation, displacing at least 65% of the total biofilm area (**Figure 6**).

#### Transcriptome analysis of P3

**Figure 7:**
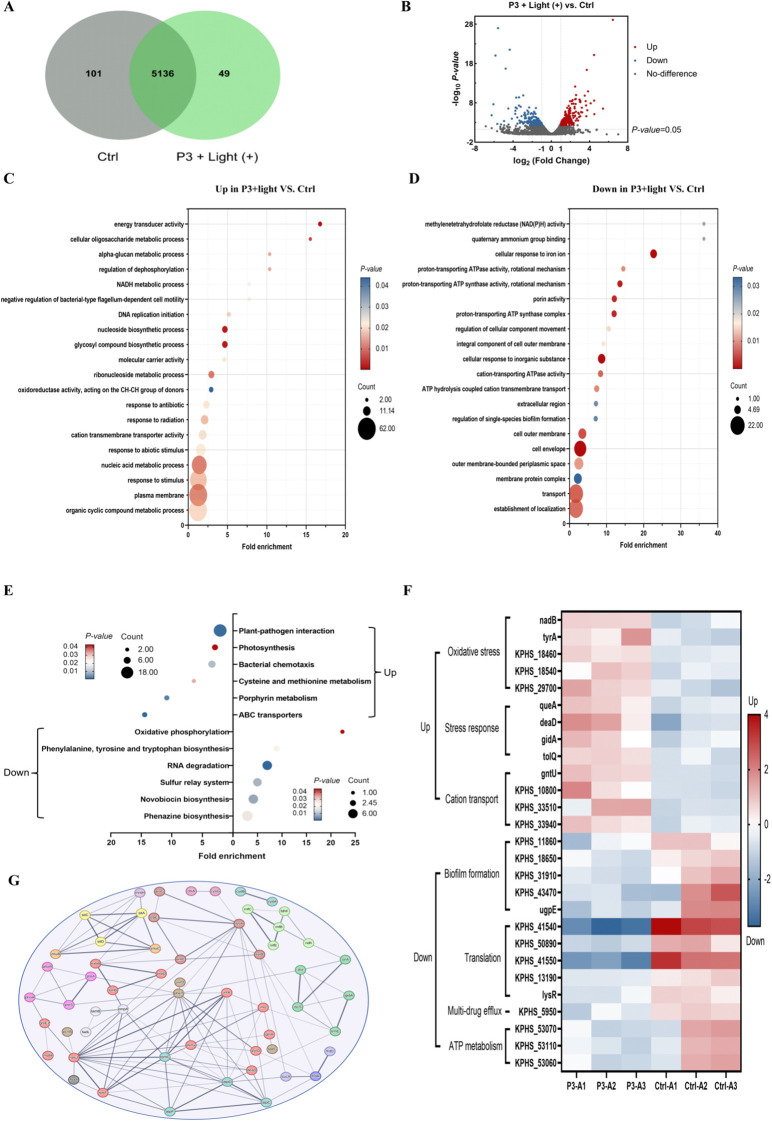
Antibacterial mechanism of P3. **(A)** Venn diagram of the DEGs in CRKP treated with P3 compared to the control group. **(B)** Volcano map of DEGs for P3 versus Control (P< 0.05, |fold change| ≥ 1.5). **(C)** Upregulated GO enrichment analysis in P3 compared with the control group. **(D)** Heatmap analysis of DEGs involved in virulence factor, resistance, localization and transport. **(E)** Enriched KEGG pathways analysis in P3 compared with the control group. **(F)** Downregulated GO enrichment analysis in P3 compared with the control group. **(G)** PPI network of the common targets.

To delineate the antimicrobial mechanism of P3, we performed RNA sequencing on CRKP treated with P3 under light exposure, with a PBS-treated group serving as the control. All samples yielded high-quality sequencing data (Q20 > 98%, Q30 > 95%), confirming data reliability for subsequent analysis (**Supplementary Table 1**). Comparative analysis identified 414 differentially expressed genes (DEGs) (Fold Change > 2, Padj< 0.05) in the P3+light group relative to the control, comprising 234 upregulated and 180 downregulated genes (**Figure 7A**, **B**).

Functional enrichment analysis of these DEGs provided mechanistic insights. Gene Ontology (GO) analysis revealed that upregulated genes were significantly associated with processes including energy transduction, suppression of flagellar motility, response to antibiotic and oxidative stress, dysfunction of cation transporters, and disruption of redox homeostasis (**Figure 7C**, **Supplementary Table 2**). Conversely, downregulated genes were enriched in functions related to cell envelope organization, regulation of biofilm formation, and various transport activities (**Figure 7D**, **Supplementary Table S3**).

Kyoto Encyclopedia of Genes and Genomes (KEGG) pathway analysis further highlighted 12 significantly enriched pathways (P< 0.05). Upregulated DEGs were mapped to pathways such as oxidative phosphorylation and sulfur metabolism, whereas downregulated DEGs were linked to ABC transporters, bacterial chemotaxis, and porphyrin metabolism (**Figure 7E**).

Focusing on key resistance determinants, we observed a pronounced upregulation of oxidative stress response genes (nadB, tyrA) and general stress mediators (queA, deaD). In parallel, a significant downregulation was evident for genes critical to biofilm formation (KPHS_11860, KPHS_18650), multidrug efflux (KPHS_5950), and ATP-dependent transport systems (**Figure 7F**). Finally, protein-protein interaction (PPI) network analysis using the STRING database, filtered for high-confidence interactions (confidence score > 0.4), distilled the complex DEG relationships into a core network of 57 intersecting target proteins (**Figure 7G**).

### *In vivo* antibacterial and wound healing performance of P3

#### Evaluation of the therapeutic efficacy of P3 in mouse skin infections

Integrated analyses demonstrate that photoactivated P3 eradicates CRKP through synergistic membrane disruption and ROS-mediated macromolecular damage, effectively disrupting biofilms and reducing bacterial viability.

**Figure 8:**
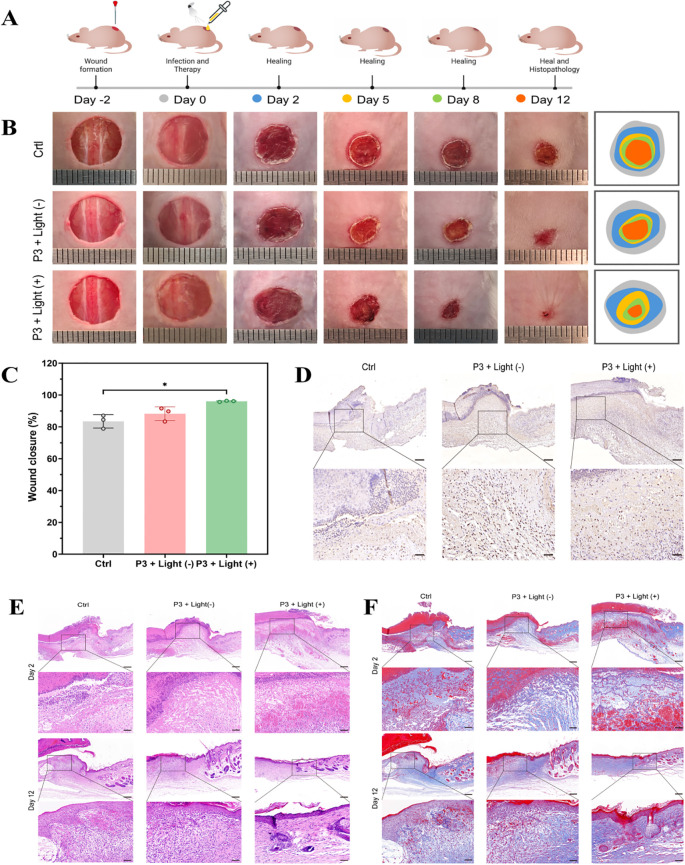
Antibacterial effectiveness of P3 for wound healing *in vivo*. **(A)** Schematic diagram for *in vivo* antibacterial treatment with various administrations. **(B)** Representative optical images of infected wound sites treated with various administrations at different time points. **(C)** Wound healing rate (%). **(D)** CD45 immunohistochemical staining images. The scale bar in the photographs is 200 μm and in the enlarged image is 50 μm. **(E)** Representative H&E and **(F)** Masson trichrome staining images of the wound tissues on day 2 and day 12. The scale bar in the photographs is 200 μm and in the enlarged image is 50 μm. *P<0.05.

In a murine skin wound model infected with CRKP, P3 plus light irradiation significantly enhanced wound closure and bacterial clearance compared to vehicle and P3-alone groups (**Figure 8A**). The P3+light group showed >25% daily wound reduction (P< 0.05), near-complete healing by day 12 (<0.5 mm²), >99% bacterial clearance (P< 0.001), and a 142% higher epithelialization rate than controls (**Figure 8B**).

P3+light promoted over 96% wound closure, significantly surpassing the control (83.5%) and P3-alone (88.2%) groups (P< 0.01; **Figure 8C**).

H&E staining revealed reduced neutrophil infiltration, active granulation, and neovascularization with P3+light by day 2, and nearly complete re-epithelialization with mature structures by day 12. Controls showed persistent inflammation and ulceration (**Figure 8D**).

Masson’s trichrome staining indicated superior collagen regeneration with dense, organized fibrils in P3+light-treated wounds by day 12, while controls had sparse, disordered collagen (**Figure 8E**).

Immunohistochemistry for CD45 confirmed markedly reduced leukocyte infiltration in P3+light wounds, indicating attenuated inflammation and a pro-healing microenvironment (**Figure 8F**).

#### Biocompatibility and biosafety assessment of P3

**Figure 9:**
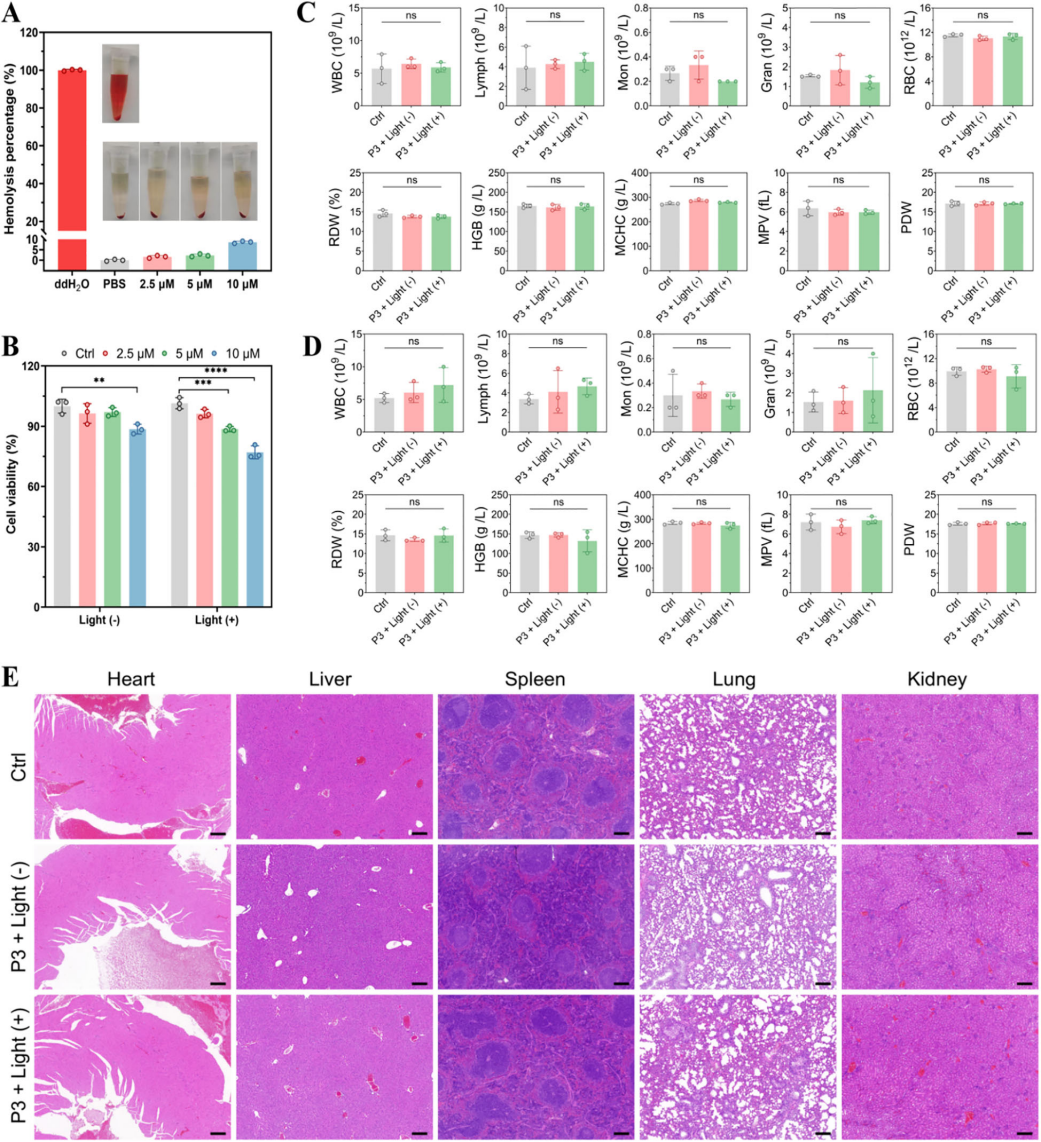
*In vivo* biocompatibility examination of P3. **(A)** hemolysis test. **(B)** cytotoxicity test. **(C)** Hematological analysis of parameters associated with oxygen-carrying capacities on the second day after administration. **(D)** Hematological analysis of parameters associated with oxygen-carrying capacities on the 12th day after administration. **(E)** H&E staining images of major organs. Scale bar is 200 μm. ns expressed P value > 0.05, with no significance.

Hemocompatibility and biosafety evaluations confirmed the systemic safety of P3. *In vitro*, 5 µM P3 induced minimal hemolysis (2.3%), below the ISO 10993–4 threshold, and preserved erythrocyte morphology (**Figure 9A**). CCK-8 assays revealed low dark cytotoxicity (viability ≥90% at 5 µM) and light-dependent toxicity within an acceptable safety margin (viability >80% at therapeutic 5 µM; **Figure 9B**).

*In vivo* hematological analysis on days 2 and 12 post-administration showed no significant alterations in immune, erythrocyte, or coagulation parameters across all groups (**Figures 9C, D**). Histopathological examination of major organs (heart, liver, spleen, lung, kidney) revealed no detectable lesions, confirming the absence of acute or chronic systemic toxicity (**Figure 9E**). These data collectively support the favorable biosafety profile and translational potential of P3.

## Discussion

CRKP is a WHO priority pathogen with high mortality rates and a major global healthcare burden (Fang et al., 2020; Chi et al., 2022). The rise of carbapenem-resistant hypervirulent strains has left few effective antibiotics (Ernst et al., 2020).

**Figure 10:**
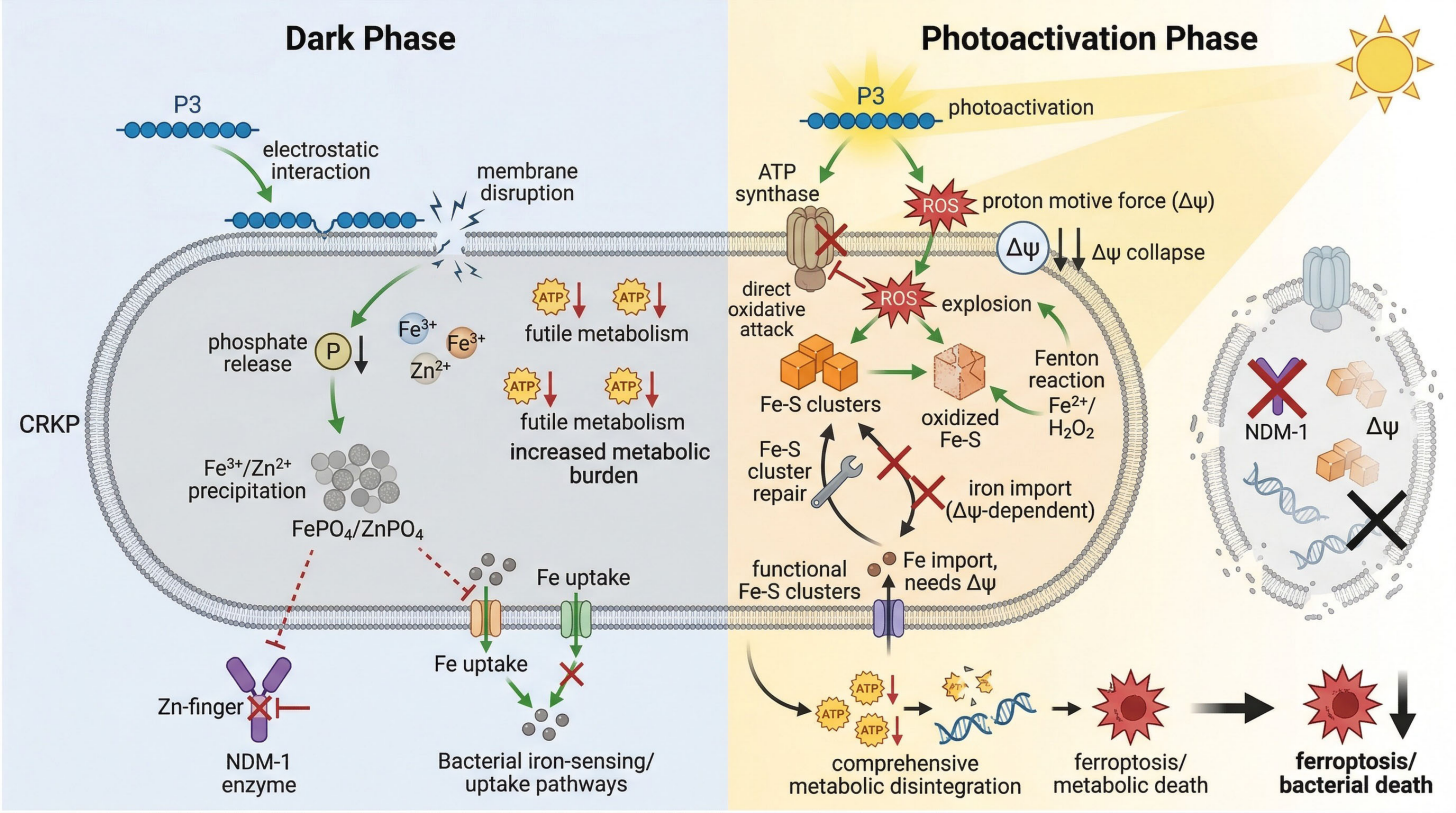
Mechanism of the antibacterial mechanism of the cationic conjugated polymer P3 against CRKP. The cationic polymer P3 eliminates carbapenem-resistant K. pneumoniae (CRKP) through a biphasic “light-controlled compensatory self-destruction” mechanism. In the dark, P3 electrostatically binds to and compromises the bacterial membrane, triggering intracellular phosphate release that precipitates Fe³^+^/Zn²^+^. This not only inactivates the Zn-finger domain of the carbapenemase NDM-1 but also misleads bacterial iron-sensing systems into futile metabolic consumption. Upon photoactivation, P3 generates a burst of reactive oxygen species (ROS) that directly attack the ATP synthase, collapsing the proton motive force (Δψ), while concurrently oxidizing Fe-S clusters in the respiratory chain to amplify oxidative damage via a Fenton reaction. The resulting energy collapse and Fe−S cluster damage create an inescapable cycle: repair of Fe-S clusters is blocked by the loss of Δψ-dependent iron import, and Δψ generation itself depends on functional Fe-S-dependent respiration. This self-reinforcing failure cascade leads to comprehensive metabolic disintegration, culminating in bacterial death through pathways such as ferroptosis, thereby circumventing conventional resistance mechanisms. This mechanism diagram was generated using [Research Mechanism Diagram AI Generator] and modified/verified by the authors to ensure scientific accuracy.

To address this, we developed a novel conjugated polymer, P3, which eliminates CRKP via a light-controlled compensatory self-destruction (CSD) mechanism (**Figure 10**). This approach disrupts bacterial metabolism with spatiotemporal precision, circumvents conventional resistance pathways, and achieves >90% antibacterial efficacy, positioning P3 as a promising candidate against multidrug-resistant CRKP.

The CSD mechanism involves a biphasic self-amplifying cascade. In the dark phase, P3 targets the membrane via electrostatic binding of its quaternary ammonium cations to phospholipids, reducing fluidity and compromising integrity (confirmed by SEM/TEM and protein leakage assays), thereby inactivating OmpA porins and displacing F-ATPase pumps (Cao et al., 2018; Effah et al., 2020; Genestra, 2007; Hong et al., 2019; Kim et al., 2019; Murata et al., 2007; Song et al., 2011). This perturbation triggers a deceptive “faux iron starvation” signal: released PO_4_³^-^ precipitates intracellular Fe³^+^/Zn²^+^ as insoluble salts, incorrectly activating the Pho and TonB systems (Bauer et al., 1988; Noinaj et al., 2010). This aberrant response upregulates phosphate transport and futile enterobactin synthesis, exhausting critical metabolites (acetyl-CoA/succinyl-CoA). Simultaneously, Zn_3_(PO_4_)_2_ formation disables the Zn-finger domain of NDM-1, inactivating the carbapenemase.

The light-activated phase executes a precision metabolic strike through a cascade of causally linked failures. Photoactivation of P3 generates a surge of reactive oxygen species (ROS), which leads to the functional collapse of ATP synthase (impaired atpD/C/F), thereby dissipating the proton motive force (Δψ) and halting cellular energy production. Concurrently, ROS oxidize Fe-S clusters in the respiratory chain, which can release iron ions and thereby promote a Fenton reaction that greatly amplifies oxidative damage. A critical, self-reinforcing repair blockade ensues. Bacterial attempts to regenerate Fe-S clusters are systematically sabotaged by a consequent triple deficit: iron may become unavailable due to precipitation, residual free Fe²⁺ is rapidly oxidized, and sulfur precursors are potentially diverted under stress. This establishes a lethal feedback loop: Fe-S repair requires iron, iron import depends on Δψ, and Δψ generation itself requires functional Fe-S-dependent respiration.(Wang, et al., 2025; Sawicki,et al., 2025).

This cascade triggers comprehensive metabolic disintegration. The failure of the hub enzyme KPHS_24560 severs the critical link between oxidative phosphorylation (ko00190) and iron-sensing (ko02020) pathways. Misallocated GroEL chaperones induce proteostatic collapse. Consequently, carbon flux is shunted from central metabolism into futile biosynthesis (e.g., enterobactin, aromatic amino acids), exhausting metabolic precursors and activating the ferroptosis pathway (ko04216). Ribosome assembly fails and futile tRNA modifications consume residual ATP. Finally, the suppression of porphyrin metabolism (ko00860) eliminates endogenous photoprotection, sealing the fatal outcome.

This orchestrated metabolic collapse directly underpins the potent phenotypic outcomes observed in our study. The combined action of initial membrane disruption and subsequent massive oxidative damage explains the observed protein leakage, bacterial death, and superior, dose-dependent biofilm eradication. CLSM visually confirmed spatiotemporally controlled biofilm decomposition from within, driven by deep P3 penetration and light-triggered activation that disrupts the EPS matrix (Liu et al., 2019).

The demonstrated properties of P3, cationic charge-driven penetration, potent light-triggered ROS generation, and a mechanism that circumvents conventional resistance, directly address the core challenges in treating localized CRKP infections. This positions P3-mediated PDT as a highly promising strategy for topical or localized intervention. Its inherent spatiotemporal control minimizes systemic exposure, making it particularly suitable for targeted applications such as in chronic wound beds, soft tissue abscesses, or as a coating for implantable devices to prevent biofilm colonization.

The robust efficacy translated into exceptional *in vivo* performance. In a murine skin wound model, P3 plus light achieved >88% wound closure, significantly surpassing all controls (P<0.05). These findings not only confirm the potent antibacterial and anti-biofilm efficacy of P3 *in vivo* but also provide direct preclinical evidence for its potential to promote healing in complex, CRKP-infected wound environments, a scenario where current options are severely limited. Histopathology revealed accelerated healing: by day 2, wounds showed neovascularization and initial collagen deposition; by day 12, they exhibited complete re-epithelialization, mature fibroblasts, and dense, organized collagen. This was supported by a potent anti-inflammatory effect, with a significant reduction in CD45+ cells by day 2.

P3’s therapeutic profile is complemented by excellent biosafety. At therapeutic concentrations, hemolysis was minimal (2.3%), and viability in NIH-3T3 fibroblasts exceeded 80% (P3+light). *In vivo*, no abnormalities were found in blood parameters or in the histology of major organs, confirming high biological safety and clinical potential.

While this study establishes the novel mechanism and compelling efficacy of P3 against CRKP, we acknowledge that direct comparative data with current standard-of-care topical agents (e.g., chlorhexidine, silver-based formulations) is crucial for defining its translational advantage. Our future work will include systematic *in vitro* and *in vivo* comparisons to quantify the superiority of P3-PDT in terms of biofilm eradication depth, speed of bactericidal action, and propensity to induce resistance relative to these conventional therapies. The initial evidence presented here, particularly the ability to penetrate and disrupt biofilms from within via a non-antibiotic mechanism, suggests a fundamental and promising difference in approach to tackling recalcitrant CRKP infections.

## Materials and methods

### Study design and experimental groups

This study was designed to evaluate the light-activatable conjugated polymer P3 for eradicating CRKP biofilms. Assessments included biofilm penetration, ROS generation, eradication efficacy, induction of compensatory self-destruction, and reduction in biofilm biomass and viability. For *in vitro* and *in vivo* experiments, the following core experimental groups were established: (1) Untreated control (phosphate-buffered saline, PBS, kept in dark), (2) Light-only control (PBS + light), (3) Polymer-only control (P3, kept in dark), and (4) P3-mediated photodynamic therapy (P3-PDT; P3 + light). Where applicable, multiple concentrations of P3 were tested. To ensure homogeneity and reproducibility of light conditions, all light irradiation experiments were performed using a light source with the same power density, and all exposed samples (e.g., wells in a plate) were irradiated equally.

### The synthetic compound P3 and the clinically isolated strains

**Figure 11:**
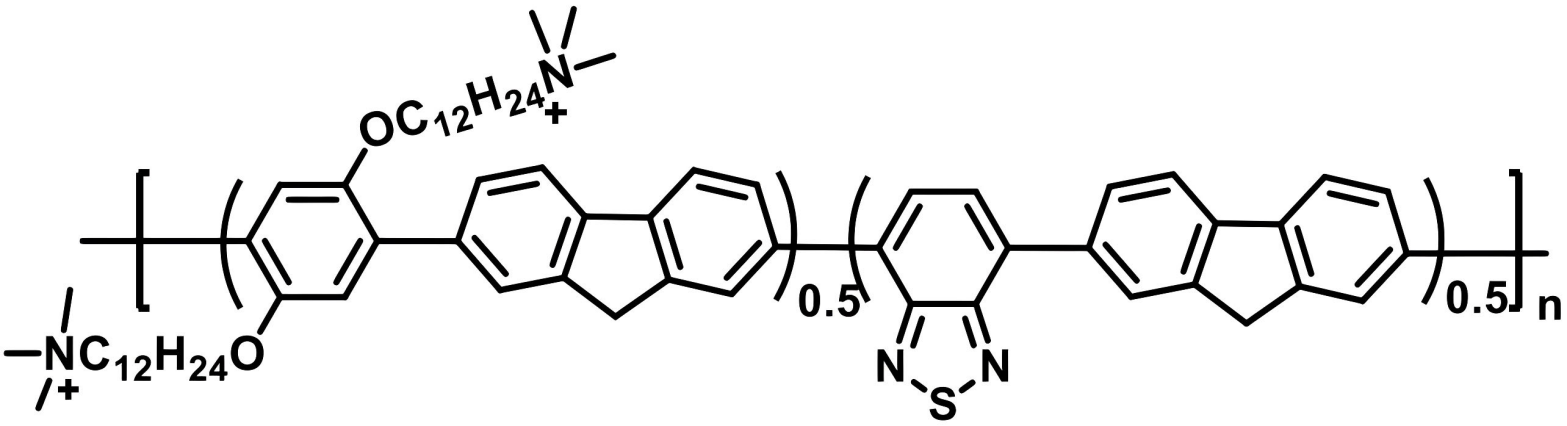
Chemical structures of P3.

P3 was provided by Professor Wang (Shanxi Medical University); synthesis and characterization were reported previously 28 (**Figure 11**). P3 is a conjugated polymer with a positive surface charge (22.9 ± 1.5 mV), a molecular weight of approximately 13.8 kDa, and good aqueous dispersibility. Its structure grants it favorable optical properties and, most notably, a high efficiency in generating reactive oxygen species (ROS). The clinical CRKP14 strain’s sequence type (ST11) and resistance gene profile (blaNDM, blaTEM, blaSHV) are based on its prior clinical characterization. .

### Light irradiation conditions

For all photodynamic treatments in this study, a white light source (400–700 nm) was used. The irradiation was performed at a power density of 25 mW/cm² (calibrated and ensured to be uniform across all samples) for a duration of 15 min. These parameters were consistently applied in all subsequent experiments involving light exposure.

### Antibacterial activity assay

As stated in the introduction, the concentration of P3 exhibited excellent antibacterial activity at 5 μm. CRKP strain was isolated from the microbiological laboratory at the Department of Pharmacy, Second Hospital of Shanxi Medical University. CRKP14 (10^8^ CFU/mL) was treated under light ((as described above) or dark conditions with P3 (2.5, 5, 10 µM) or PBS in EP tube. After incubation (37 °C, 20 min) and light exposure where applicable, serial dilutions were plated onto solid TSB agar plates. Colonies were counted after 16 h of incubation at 37 °C. The viability rate (VR) was calculated according to the following equation:


$$\text{VR}=\frac{\text{C}}{{\text{C}}_{0}}\times 100\%$$

where C is the colony forming unit (cfu) of the experimental groups, and C_0_ is the cfu of the control group without polymer treatment in the dark.

### Membrane disruption and morphology

Bacteria from four treatment groups were processed for SEM/TEM. For scanning electron microscopy (SEM), samples were fixed with 2.5% glutaraldehyde overnight at 4°C, dehydrated through a graded ethanol series (30%, 50%, 70%, 80%, 90%, and 100%), and critically point dried. For transmission electron microscopy (TEM), fixed samples were post-fixed with 1% osmium tetroxide for 2 h, dehydrated, and embedded in epoxy resin. Ultrathin sections (70–90 nm) were double-stained with uranyl acetate and lead citrate.

### Protein leakage assay and ROS detection

The supernatant from treated bacteria was collected by centrifugation. The extracellular protein concentration was quantified using a bicinchoninic acid (BCA) protein assay kit, measuring the absorbance at 562 nm against a standard curve.

Bacterial suspensions were loaded with 10 µM 2′,7′-dichlorodihydrofluorescein diacetate (DCFH-DA) at 37 °C for 30 min in the dark. After washing, the bacteria were treated with PBS or 5.0 µM P3 under light or dark conditions for 20 min. Fluorescence intensity was measured with a microplate reader (excitation/emission: 488/525 nm).

### Biofilm assays

Biofilm Formation and Treatment: CRKP biofilms were grown in 24-well plates (48 h, 37 °C) containing TSB supplemented with 5% glucose for 48 h at 37 °C, with the medium replaced at the 24-hour mark. Mature biofilms were treated with P3 (2.5, 5.0, or 10.0 µM) or PBS for 30 min at 37 °C, followed by light irradiation (25 mW/cm², 15 min) or dark incubation. The light source was calibrated to deliver uniform power density across all exposed wells.Biomass Quantification (Crystal Violet Assay): Treated biofilms were washed, fixed with methanol for 15 min, stained with 0.5% crystal violet for 10 min, and solubilized with 2% acetic acid. Absorbance at 570 nm was measured. The anti-biofilm efficacy was calculated as: (ODcontrol - ODexperimental)/ODcontrol × 100%.

Biofilm Viability and Polymer Penetration (CLSM): Biofilms treated with 10.0 µM P3 or PBS ± light were stained using a Live/Dead BacLight Bacterial Viability Kit. Briefly, biofilms were incubated with Calcein-AM solution for 30 min at 37 °C, washed, then incubated with propidium iodide solution for 15 min at room temperature, protected from light. The intrinsic fluorescence of P3 was simultaneously captured. Three-dimensional confocal laser scanning microscopy (CLSM) was used to visualize biofilm viability and the penetration profile of P3.

### Transcriptomic analysis of P3-mediated photodynamic effects on CRKP

Total RNA was extracted from CRKP14 cultures treated with P3+light or PBS (control) using TRIzol reagent. Ribosomal RNA was depleted using the RiboMinus™ Bacteria Kit. Strand-specific cDNA libraries were prepared with the NEBNext^®^ Ultra™ II RNA Library Prep Kit and sequenced on an Illumina NovaSeq 6000 platform (2×150 bp paired-end). Quality-filtered clean reads were aligned to the *Klebsiella pneumoniae* reference genome (ASM100000v2). Differential gene expression analysis was performed using DESeq2 (version 1.34.0), with thresholds set at |log_2_(fold change)| ≥ 1 and false discovery rate (FDR)< 0.05. Gene Ontology (GO) and Kyoto Encyclopedia of Genes and Genomes (KEGG) pathway enrichment analyses were conducted on the differentially expressed genes (DEGs). Hierarchical clustering heatmaps were generated to visualize DEG expression profiles.

### *In vivo* studies

All animal experiments were approved by the Ethics Committee (SYXK (JIN) 2024-0001, No. 2024086).

Sample Size and Grouping: Based on preliminary data indicating a large effect size (Cohen’s d > 1.5) for wound healing rate, an *a priori* power analysis (α = 0.05, power = 0.80) suggested a minimum of n=5 per group. To account for potential attrition, a total of 18 male Balb/c mice (6–8 weeks old, 20–22 g) were randomly allocated into four groups (n=6 each): (1) PBS + dark, (2) 5 µM P3 + dark, (3) 5 µM P3 + light (P3-PDT).

Wound Infection and Treatment: Mice were anesthetized, and a full-thickness circular wound (approximately 1 cm²) was created on the shaved dorsum. Wounds were inoculated with 100 µL of CRKP14 suspension (10^6^ CFU). After 48 h to establish infection, wounds were topically treated with 50 µL of 5 µM P3 or PBS. The P3-PDT and light-only control groups were immediately irradiated with white light (400–700 nm, 25 mW/cm²) for 15 min. All wounds were covered with a sterile hydrogel dressing.

Outcome Measures: Wound areas were photographed and measured daily. The wound healing rate on day n was calculated as: (1 − A_n_/A_0_) × 100%.

where A_0_ is the initial wound area and An is the wound area measured on day n.

On days 2 and 12 post-treatment, mice (n=3 per time point per group) were euthanized for tissue and blood collection.

### Histological analysis

Wound tissue samples were fixed in 4% paraformaldehyde for 24 h at 4 °C, dehydrated through a graded ethanol series, cleared in xylene, and embedded in paraffin. Sections (4 µm thick) were cut using a microtome (Leica RM2235). These slices were then stained using Hematoxylin and Eosin (H&E), Masson staining, and CD45 immunohistochemistry (IHC); following staining, the sections were visualized under bright-field microscopy.

### Biosafety assessments

The cytocompatibility of P3 was evaluated in mouse embryonic fibroblast cells (NIH-3T3). Cells were seeded in 96-well plates at a density of 1×104 cells/well and cultured in DMEM supplemented with 10% FBS at 37 °C under 5% CO_2_ until reaching approximately 70% confluency. The medium was then replaced with treatment solutions: DMEM alone (control), P3 at various concentrations (2.5, 5, and 10 μM), or P3 at the same concentrations followed by light exposure. After 18 hours of incubation, cells were washed twice with PBS and incubated with 10% (v/v) CCK-8 reagent in serum-free DMEM for 2 hours. Absorbance at 450 nm was measured to determine cell viability. The cell survival rate was calculated using the formula: Cell survival rate (%) = (ODexperimental - ODblank)/(ODcontrol - ODblank) × 100%; ODblank represents the absorbance value of the CCK-8 solution in DMEM without cells.

### Hemolysis assay

Mouse red blood cells (RBCs) were washed and diluted to a 10% (v/v) suspension in PBS. The suspension was incubated with various concentrations of P3, PBS (negative control), or deionized water (positive control) at 37 °C for 2 h. After centrifugation, the absorbance of the supernatant was measured at 540 nm. Hemolysis rate was calculated as follows: Hemolysis (%) = (ODexperimental - ODblank)/(ODcontrol - ODblank) × 100%. Each experimental condition was performed in triplicate to ensure accuracy and reliability.

### Serum biochemistry and organ histopathology

Blood samples collected on days 2 and 12 were analyzed for routine hepatic and renal indicators using an automated biochemical analyzer (Type 7170, Hitachi, Japan). Major organs (heart, liver, spleen, lungs, and kidneys) were harvested, fixed, sectioned, and stained with H&E for histopathological examination.

### Statistical analysis

Data are expressed as the mean ± SD. Statistical analyses were conducted using GraphPad Prism software (v9.0), employing one-way ANOVA followed by Tukey’s *post hoc* test for multiple comparisons. Statistical significance was set at *p< 0.05, **p< 0.01, ***p< 0.001, and ****p< 0.0001.

## Data Availability

The original contributions presented in the study are included in the article/**Supplementary Material**. Further inquiries can be directed to the corresponding author.
