# Correction: A conjugated polymer antimicrobial agent triggers metabolic cascade-mediated killing of carbapenem-resistant *Klebsiella pneumoniae*

**DOI:** 10.3389/fcimb.2026.1859021

**Published:** 2026-05-05

**Authors:** Shunyun Wang, Song Wang, Lin Chen, Binbin Li, Chuangxin Zhang, Donghong Yin, Jinju Duan

**Affiliations:** 1 Department of Pharmacy, Second Hospital of Shanxi Medical University, Taiyuan, Shanxi, China; 2 School of Pharmacy, Shanxi Medical University, Taiyuan, Shanxi, China; 3 School of Chemistry and Chemical Engineering, Shanxi University, Taiyuan, Shanxi, China

**Keywords:** activity-tunable, compensatory, conjugated, CRKP, metabolic hijacking, polymer, self-destruction, spatiotemporal control

The order of authors in the author list of the published paper was erroneously given as:

“Shunyun Wang, Song Wang, Lin Chen, Binbin Li, Chuangxin Zhang, Donghong Yin^*^ and Jinju Duan^*^”

The correct author list reads:

“Shunyun Wang^†^, Song Wang^†^, Lin Chen, Binbin Li, Chuangxin Zhang, Donghong Yin^*^ and Jinju Duan^*^”

^†^These authors have contributed equally to this work

Author “Song Wang” was erroneously assigned to affiliation “^2^Department of Pharmacy, Second Hospital of Shanxi Medical University, Taiyuan, Shanxi, China”.

This affiliation has now been removed for author “Song Wang”.

Affiliation “^1^School of Pharmacy, Shanxi Medical University, Taiyuan, Shanxi, China” was omitted from the paper. This affiliation has now been added for author “Song Wang”.

The original version of this article has been updated.

