# Supplementary material for: A conjugated polymer antimicrobial agent triggers metabolic cascade-mediated killing of carbapenem-resistant Klebsiella pneumoniae
Source: Front Cell Infect Microbiol. 2026 Apr 16;16:1796479. doi: 10.3389/fcimb.2026.1796479 (PMC13128601; doi:10.3389/fcimb.2026.1796479)
Supplement: Supplementary file 1 [file DataSheet1.pdf]

**Table S1. Quality Indicators of Sequencing Data**

| Samplename | Rawreads | Cleanreads | Rawbases | Cleanbases | Q20 (%) | Q30 (%) | GCratio (%) |
|---|---|---|---|---|---|---|---|
| Ctrl-A1 | 59709746 | 56633398 | 9.0G | 6.6G | 99.30 | 97.43 | 54.47 |
| Ctrl-A2 | 61412754 | 58237714 | 9.2G | 6.9G | 99.37 | 97.58 | 54.47 |
| Ctrl-A3 | 65424188 | 62205004 | 9.8G | 7.3G | 99.36 | 97.54 | 54.50 |
| P3-A1 | 71454136 | 69910678 | 10.7G | 10.2G | 98.64 | 95.71 | 53.80 |
| P3-A2 | 59891300 | 58130670 | 9.0G | 8.4G | 98.36 | 95.61 | 54.36 |
| P3-A3 | 61216008 | 59410320 | 9.2G | 8.6G | 98.53 | 95.83 | 54.49 |

**Table S2. Statistical Table of Different GO Functions (Up-regulated)**

| GO | Count |
|---|---|
| biological process | 65 |
| cellular component | 8 |
| molecular function | 20 |

**Table S3. Statistical Table of Different GO Functions (Down-regulated)**

| GO | Count |
|---|---|
| biological process | 57 |
| cellular component | 19 |
| molecular function | 36 |
